# Development and implementation of a point of care ultrasound curriculum at a multi-site institution

**DOI:** 10.1186/s13089-021-00214-w

**Published:** 2021-02-21

**Authors:** Frances M. Russell, Audrey Herbert, Robinson M. Ferre, Bita Zakeri, Valerie Echeverria, Dina Peterson, Paul Wallach

**Affiliations:** 1 Department of Emergency Medicine, Indiana University School of Medicine, 720 Eskenazi Ave, Fifth Third Faculty Office Building, 3rd Floor Emergency Medicine Office, Indianapolis, IN 46202 USA; 2 Education and Continuing Medical Education, Indiana University School of Medicine, Indianapolis, IN USA; 3 Indiana University School of Medicine, Indianapolis, IN USA; 4 Department of Internal Medicine, Office of the Dean, Indiana University School of Medicine, Indianapolis, IN USA

**Keywords:** Point of care ultrasound, Medical education, Medical students, Integrated curriculum, Multi-site campus

## Abstract

In 2014, over 60% of medical schools were incorporating point of care ultrasound (POCUS) into their curriculum. Today, over 6 years later, many more schools are teaching POCUS or are in the planning stages of implementing a POCUS curriculum. In 2019, the AAMC reported that 53 schools or over one-third of US medical schools have multi-site campuses for undergraduate medical education. Implementation of a POCUS educational initiative at a multi-site campus presents unique challenges for teaching a uniform curriculum statewide. This article will discuss the POCUS curriculum and implementation process at a large multi-site institution.

## Background

Point of care ultrasound (POCUS) is a limited ultrasound examination used by the clinician at the bedside to answer a focused clinical question, guide treatment decisions and/or for procedural guidance [1–4]. POCUS in undergraduate medical education continues to expand with over 60% of medical schools incorporating this training into their curriculum [5, 6]. In 2018, Indiana University School of Medicine began to plan and implement a 4-year longitudinal POCUS curriculum to train medical students. Indiana University School of Medicine is currently the largest medical school in the United States, with over 360 students per year spread across 9 separate campuses throughout the state of Indiana. The size and geographic distance of the campuses presented unique challenges for teaching POCUS statewide. These same challenges are faced by many multi-site schools across the nation. In this manuscript we discuss the POCUS curriculum and implementation process at a large multi-site institution.

## Curriculum development and implementation

POCUS, like many other clinical skills, can be broken down into smaller sub-competencies that can be both individually taught and assessed. These have previously been described for POCUS [7, 8] and include: (1) understanding the indications for a POCUS exam; (2) having the ability to operate an ultrasound machine and obtain interpretable images; (3) recognizing basic pathologic conditions and differentiating those from normal; (4) successfully applying the results of a POCUS exam to a patient’s clinical care. The purpose of our curriculum was two-fold: (1) to create a scaffolding to augment learning in anatomy and physical exam; (2) to use POCUS as a clinical skill to aid in diagnosis and procedural assistance.

Integrating this curriculum within 4 years of undergraduate medical education curriculum allowed for cognitive scaffolding across theoretical and practical knowledge and skills learned, ultimately enhancing traditional learning and leading to improved diagnostic and procedural skills. Components of the curriculum included online modules, proctored hands-on scanning sessions, open lab time with peer teaching and assessments.

### Instructional material and modules

**Fig. 1:**
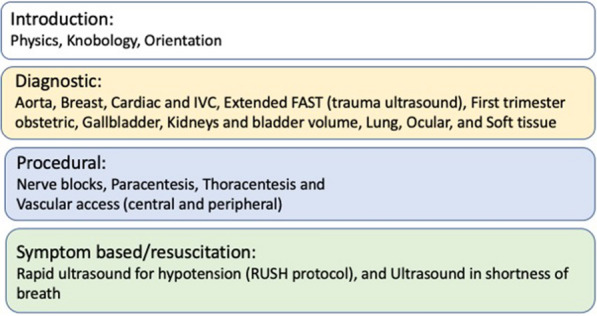
POCUS Modules

To provide didactic instruction, an online POCUS course was developed that consisted of ultrasound modules that corresponded to instructional material being taught at different time points within the undergraduate medical curriculum. The initial phase of POCUS implementation included sixteen unique modules made available to students prior to hands-on instruction, see Fig. 1.

These modules were developed to augment existing educational objectives, as well as to give students the POCUS foundation and skills necessary to enhance their clinical practice. Modules were divided into diagnostic, procedural and symptom-based categories. Diagnostic modules were divided into beginner and advanced sections. The beginner level of each module focused on anatomic structures, image acquisition and interpretation; the advanced level focused on pathology, interpretation, clinical integration and treatment. The different levels of learning in each module were intentionally designed to increase cognitive scaffolding across the curriculum, leading to deeper levels of understanding of anatomy, pathology and treatment. In the first year of curriculum integration, assessment of 147 students found that 73% watched the online didactics prior to the hands-on scanning session for anatomy, while 27% did not complete the pre-lab assignments.

### Hands-on instruction

Proctored hands-on POCUS instruction was integrated into the curriculum throughout multiple classes including anatomy, physical examination skills, clerkships and emergency medicine. During anatomy and physical examination courses, for first and second year students, POCUS labs were required. Depending on the resources available at each campus these labs were held either five times over the year with the content mirroring what the students were learning during dissection or were combined into a single lab with training for the same amount of time.

Survey data collected from 100 first year medical students after anatomy hands-on POCUS instruction, found that 71% agreed that the pre-lab modules helped prepare them to manipulate the US equipment, while 11% disagreed. Eighty-seven percent reported increased confidence with identifying the gallbladder and performing a cardiac exam after the lab, while 1% felt it worsened their ability. The majority of students commented that they “would have liked more time” in the curriculum to learn ultrasound. Survey data collected from 108s year students after their physical examination hands-on POCUS instruction, found that 91% agreed that the pre-lab modules helped prepare them to manipulate the US equipment, while 2% disagreed. Ninety-eight percent reported increased confidence with performing a FAST and cardiac exam after the lab. Two percent were neutral and none felt that the lab worsened their ability.

During clerkships and emergency medicine rotations for third and fourth year students, hands-on instruction was required during orientation and available throughout the rotation depending on the site. During hands-on labs students performed peer-to-peer scanning or used a standardized patient.

### Open scanning labs

Open scanning labs were optional and offered to students as an additional opportunity to gain hands-on experience and to further review the module content. Open labs were promoted through the school’s electronic newsletter and intranet site. For first and second year medical students labs were held every other week at each campus and scheduled to coincide with free-time built into the students’ curricular program. These sessions were proctored by a registered diagnostic medical sonographer or a sonography student to ensure expert guidance. For third and fourth year medical students labs were held once a month and were not proctored.

### Assessments

We started assessments during the second year of implementation. Within the anatomy course we added post-lab knowledge assessment questions to the already existing end of block examinations. These assessments included true/false and multiple choice questions, and covered anatomic structures on POCUS images and image acquisition. Assessments have yet to be added to the physical examination course and clerkships; however, we plan on adding assessments in an objective structure clinical examination (OSCE) format and using simulation for identification of pathology and incorporation into clinical practice.

### Feedback

Feedback was imperative for successful implementation of this educational initiative. We regularly collected feedback from both students and faculty to continually adjust and improve the experience for both. Feedback was mainly collected through post-lab surveys and directly from students (representative of years 1–3) who sat on the POCUS committee. Faculty feedback regarding the design of the curriculum, online modules and training sessions was critical to the development and integration POCUS into the existing curriculum.

### Equipment

Physical space and POCUS equipment were key components in planning a longitudinal POCUS experience across all 9 campuses. Diagnostic POCUS machines are expensive and physical space to house them can be challenging to find. Ancillary equipment (e.g., ultrasound gel, tables for models to lay on, cleaning solution, towels for draping, etc.) required for hands-on scanning labs was also an important consideration in budgeting and planning.

After exploring different types of POCUS equipment, it was evident that handheld POCUS equipment offered several advantages over larger cart-based imaging systems. Handheld POCUS equipment could be purchased at a fraction of the cost and secure storage of these devices required little space. Handheld equipment could also be checked out to individual users for several days at a time, thus allowing equipment to be used outside of dedicated lab time.

Taking into consideration quality, cost and diversity of the handheld devices equal numbers of Butterfly iQ (Butterfly Network, Inc, Guilford, CT, USA) and Philips Lumify (Philips North America Corp, Andover, MA, USA) devices were purchased. Having two different machines provided variation in POCUS educational tools for learners. Butterfly iQ uses CMET technology, which enables a single probe the ability to image both superficial and deep structures. We purchased Lumify phased array probes to image cardiac, lung and abdomen.

### Simulators

Simulators were bought with the idea of teaching procedural guidance, introducing students to pathology on POCUS and learning clinical integration. For this reason Blue Phantom (CAE, Inc, Montreal, Quebec, Canada) and SonoSim LiveScan (SonoSim, Inc, Santa Monica, CA) were purchased for all campuses. During the Anesthesia clerkship a hands-on ultrasound guided vascular access lab was taught using the vascular models. These branched vessel ultrasound phantoms served as low fidelity simulators and were reusable.

SonoSim LiveScan was incorporated into the physical examination course hands-on lab for second year students in a case based format to illustrate pathology and for students to get comfortable incorporating POCUS into clinical practice.

### Location and check out procedure for equipment

Selection of an optimal space to both store equipment and train learners across all 9 campuses in an equitable manner was important. We chose the simulation center, medical library and hospital space designated for teaching. The space used varied by campus based on availability, size and accessibility for students. Handheld POCUS devices and tablets were securely stored in locked cabinets at each site along with other necessary supplies such as gel and cleaning solution.

In addition to scheduled POCUS events, faculty and students could check-out equipment to practice scanning. A reservation software program was used to reserve and track equipment and track the length of check-out time. Detailed instructions for checking out equipment were disseminated through the school using a newsletter and intranet site.

### Coordination with stakeholders

To implement a longitudinal POCUS curriculum at a multi-site campus, key sponsors, educators and course directors were initially identified across the state. Under the direction of the Executive Associate Dean for Educational Affairs a POCUS committee was created to coordinate and communicate with key stakeholders. Members of this committee included faculty of diagnostic radiology, ultrasound-trained emergency medicine faculty, radiology faculty, medical students from each phase of education and a program manager.

The POCUS committee developed, piloted and subsequently oversaw a phased implementation of the 4-year curriculum. To incorporate the POCUS modules within the existing courses, the committee worked collaboratively with course directors and local and regional deans to develop a plan for deployment of the curriculum within the targeted courses at all sites. Communication at multiple levels was crucial in synchronizing the integration of POCUS across the state.

In addition, a point of care ultrasound advisory committee was developed at the onset, and members consisted of phase Deans, multidisciplinary ultrasound trained faculty, and simulation center leadership. The initial focus of this group was to coordinate implementation strategies across the state, disseminate information widely, give feedback and collaborate with key stakeholders. The committee was successful in creating a space to network and collaborate with invested faculty from across multiple departments. However, the committee became less active through the implementation process. The plan, as the curriculum is further improved and expanded, is that the advisory committee will stay involved.

### Finding local champions

Local champions, with expertise in POCUS and teaching, spearheaded training and implementation of the curriculum on each campus. We identified regional campus champions through regional deans’ recommendation and through individuals who nominated themselves. Student enthusiasm for this initiative, availability of resources, along with support of the regional campus deans presented an inviting opportunity for faculty to get involved. These faculty worked directly with the central POCUS planning team. Regional champions locally guided faculty and student training, phased implementation of POCUS modules within the curriculum and ensured an equitable student learning experience across all campuses.

### Faculty education

Lack of faculty skill and confidence in performing and teaching POCUS remains a large barrier to implementation of a longitudinal curriculum and holds true especially at a multisite campus, where instruction is bound geographically [5, 6]. We held multiple continuing medical education (CME) accredited training sessions for faculty in an effort to increase POCUS utilization and medical student teaching by varied faculty across the school. These sessions were promoted over email and offered during different days and times of the week to increase the number of opportunities for participants to attend. Almost 80 faculty across multiple specialties completed a 6-h POCUS CME training workshop. These workshops included didactics and hands-on instruction covering multiple exam types. A primary goal of these workshops was to increase confidence and knowledge with POCUS and give faculty the tools to teach POCUS to medical students. Data analysis of 78 faculty participants found that a majority of the faculty were novice to ultrasound (68%). By assessing confidence, knowledge and skill we found that after a short training faculty had increased confidence with using and teaching POCUS (*p* < 0.01), showed improved knowledge from 50 to 86% (*p* < 0.01) and were able to correctly identify anatomic structures with ultrasound with good image quality [9].

### Interprofessional

While faculty training was being implemented, senior sonography students with an interest in POCUS, ultrasound fellows and fourth year medical students who had previously completed a 1-month elective in POCUS served as a critical resource for medical student training. Sonography students served as proctors during required and independent hands-on labs.

Ultrasound fellows and fourth year medical students, likewise, served as instructors during required hands-on labs. Lab facilitator guides were created, which included objectives of the lab, required images to obtain and tips for acquiring images. These, in addition to the modules, were available online and reviewed by proctors prior to lab.

### Placement of POCUS within the curriculum

**Fig. 2:**
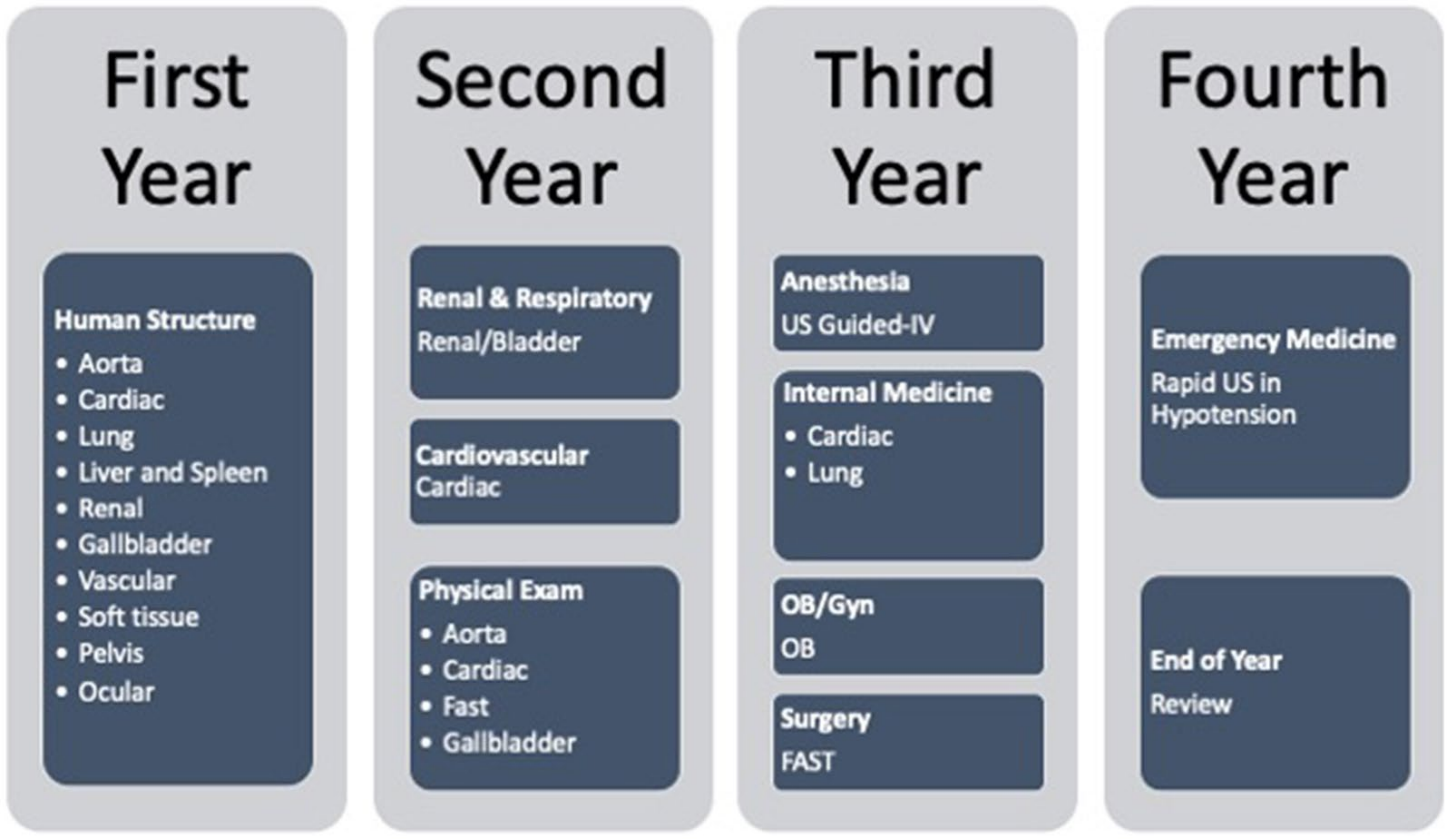
Undergraduate medical education curriculum showing where POCUS modules were implemented, specifically in which year of schooling and in what course

Sixteen modules were included in the initial POCUS curriculum. The content of these modules were compared to the existing curriculum to decide, where each module would best fit. Anatomy and physical examination courses were identified as two subject areas, where POCUS would augment the curriculum. POCUS modules were divided into beginner and advanced. Beginner modules included anatomy and image acquisition and these were incorporated into the anatomy course. For these modules we focused on content that would subsequently be used in clinical POCUS exams. For example, dedicated modules that focused on anatomic structures identifiable on cardiac ultrasound were developed. Students were able to use POCUS to visualize anatomic structures in real-time, using POCUS views that would later be incorporated into the clinical evaluation of patients. This served to not only supplement existing curriculum, but to also serve as a foundation for future POCUS education see Fig. 2.

During the clinical phase of the curriculum, placement of POCUS modules were paired to the respective clerkship, where they were most applicable. For example, first trimester obstetric ultrasound was chosen for implementation in the obstetrics and gynecology clerkship, extended focus assessment of sonography in trauma was implemented in the surgery clerkship, etc. As many faculty in these specialties already use targeted POCUS in their practice, existing faculty skill and knowledge base could, therefore, be capitalized.

### Piloting curriculum

To evaluate the feasibility of implementation and achievement of educational objectives, we piloted the POCUS curriculum at one regional campus in the anatomy course. Three sessions were held throughout the year using asynchronous viewing of online modules and hands-on instruction to deliver content. Students were then surveyed regarding implementation of POCUS within anatomy. Survey data of 40 students after course implementation found that 100% of students indicated that ultrasound enhanced their understanding of anatomy, and found adding ultrasound to anatomy lab to be beneficial to their learning. We found the piloted curriculum to be feasible and well received by both faculty and students. The following year it was implemented across all nine campuses.

### Phased implementation

Due to resource limitations, including time in current curriculum and lack of trained faculty, POCUS was implemented using a phased approach. We started by integrating POCUS into anatomy, clerkships and emergency medicine. These areas were targeted as they required the least amount of training and resources, or already had a POCUS component. In addition, independent scanning labs were offered throughout the year. The following year we expanded to the physical examination and pathophysiology courses. We also added in knowledge assessments to the anatomy course. Using a phased approach we were able to ensure an equivalent POCUS experience for students across all campuses. Additionally, it allowed more faculty to gain more exposure to POCUS without the curriculum becoming overwhelming.

### Student consent form

Consent was required from a student if they volunteered to serve as an ultrasound model at any time. An electronic consent form was developed specifically for this use. All students were asked to accept or decline participation as a model for other students or learners in an educational setting. The form was placed within the Medical Student Administration System, which is a student portal. All first-year students were instructed to complete the consent form during their anatomy course. POCUS hands-on lab facilitators verified consent completion prior to any hands-on scanning. Students could change consent status at any time.

### Development of an US Website

The POCUS website was created with the intent to have all information stored in one central location that could be easily disseminating in a timely fashion to all faculty, staff and medical students. The website houses modules, lab facilitator guides, equipment check-out procedures, open lab times and consent forms.

## Amendments and future directions

Implementing a POCUS curriculum across a large multi-site medical school has been successful, in large part because of support from the executive leadership, collaboration with key stakeholders and the universal positive reception from medical students. Each of these three elements have been and remain critical for success. Competition for curricular space and time within the first 2 years was particularly challenging. Our staged implementation approach with centrally created online didactics put less strain on basic science faculty and created time and space for them to incorporate this new content into their courses. Indeed, due to the logistical constraints of a multi-site medical school, the initial aims of curricular integration were focused on creating material, training faculty and finding time within the existing curriculum. Creation of didactic content by the POCUS committee and finding local experts to assist with hands-on instruction proved vital to successful implementation. However, we believe, the ultimate success and longevity of the curriculum will be measured by the uptake and ownership from course directors and local educators, and not the central POCUS committee.

By design, assessment of student’s POCUS knowledge and hands-on skill were not part of the initial implementation of the curriculum. Incorporating knowledge-based assessment into pre-existing testing strategies, such as pre-existing block exams, has and will continue to be important for integration. Skill-based assessments are being planned for clinical rotations with the plan to incorporate hands-on POCUS assessment into pre-existing OSCEs that occur at the end of third year. While standardized patients do not have pathology, students will be required to perform the appropriate POCUS exam, identifying to examiners the anatomy and location of the pathologic findings germane to the case. They will then be given images of pathology for the case, asked to interpret the images and apply them to the patient in the exam scenario. For pre-clinical years, this coming academic year we will begin skill-based assessment as students submit images for review, which they will upload to our learning management system and graded using a simple rubric.

Finally, while not focused on the individual student, we plan to introduce competitive gamification of POCUS during the fourth year, similar to what has previously been described [10]. Subject material would be comprehensive from all 4 years of POCUS instruction. Teams of students would compete at stations designed to assess knowledge and or specific skill.

While national curricular standards do not exist for POCUS integration, professional organizations like Society of Ultrasound in Medical Education (SUSME) allow for sharing of ideas and resources for schools looking to begin a POCUS curriculum. Content sharing through MedED Portal and Canvas Commons are additional ways we hope to share our experience and content.

## Conclusion

There are many unique challenges to implementing a longitudinal POCUS curriculum at a multi-site institution. These same barriers are faced by many schools across the country. This report describes an approach using a phased implementation that has been found to be successful, and may serve as a reference for POCUS curriculum design.

## Data Availability

Not applicable.
